# COSMOS: COmparing Standard Maternity care with One-to-one midwifery Support: a randomised controlled trial

**DOI:** 10.1186/1471-2393-8-35

**Published:** 2008-08-05

**Authors:** Helen L McLachlan, Della A Forster, Mary-Ann Davey, Judith Lumley, Tanya Farrell, Jeremy Oats, Lisa Gold, Ulla Waldenström, Leah Albers, Mary Anne Biro

**Affiliations:** 1 Mother and Child Health Research, La Trobe University, 324-328 Little Lonsdale St, Melbourne, Australia; 2 Division of Nursing and Midwifery, La Trobe University, Bundoora, Australia; 3 Royal Women's Hospital, Locked Bag 300, Grattan St and Flemington Rd, Parkville, Australia; 4 Consultative Council on Obstetric and Paediatric Mortality and Morbidity, Department of Human Services, 50 Lonsdale St, Melbourne, Australia; 5 Health Economics Unit, School of Health and Social Development, Deakin University, 221 Burwood Highway, Burwood, Australia; 6 Department of Nursing, Karolinska Institutet, Stockholm, Sweden; 7 College of Nursing, University of New Mexico, Albuquerque, New Mexico

## Abstract

**Background:**

In Australia and internationally, there is concern about the growing proportion of women giving birth by caesarean section. There is evidence of increased risk of placenta accreta and percreta in subsequent pregnancies as well as decreased fertility; and significant resource implications. Randomised controlled trials (RCTs) of continuity of midwifery care have reported reduced caesareans and other interventions in labour, as well as increased maternal satisfaction, with no statistically significant differences in perinatal morbidity or mortality. RCTs conducted in the UK and in Australia have largely measured the effect of *teams *of care providers (commonly 6–12 midwives) with very few testing *caseload *(one-to-one) midwifery care. This study aims to determine whether caseload (one-to-one) midwifery care for women at low risk of medical complications decreases the proportion of women delivering by caesarean section compared with women receiving 'standard' care. This paper presents the trial protocol in detail.

**Methods/design:**

A two-arm RCT design will be used. Women who are identified at low medical risk will be recruited from the antenatal booking clinics of a tertiary women's hospital in Melbourne, Australia. Baseline data will be collected, then women randomised to caseload midwifery or standard low risk care. Women allocated to the caseload intervention will receive antenatal, intrapartum and postpartum care from a designated primary midwife with one or two antenatal visits conducted by a 'back-up' midwife. The midwives will collaborate with obstetricians and other health professionals as necessary. If the woman has an extended labour, or if the primary midwife is unavailable, care will be provided by the back-up midwife. For women allocated to standard care, options include midwifery-led care with varying levels of continuity, junior obstetric care and community based general medical practitioner care. Data will be collected at recruitment (self administered survey) and at 2 and 6 months postpartum by postal survey. Medical/obstetric outcomes will be abstracted from the medical record. The sample size of 2008 was calculated to identify a decrease in caesarean birth from 19 to 14% and detect a range of other significant clinical differences. Comprehensive process and economic evaluations will be conducted.

**Trial registration:**

Australian New Zealand Clinical Trials Registry ACTRN012607000073404.

## Background

Continuity of carer in the provision of maternity care has been strongly recommended and encouraged in Victoria and throughout Australia. The Victorian Department of Human Services (DHS) released a policy document "Future directions for Victoria's maternity services" [1] in June 2004 which endorsed and promoted the expansion of public models of maternity care that offer continuity of *carer*. Many hospitals have responded by introducing caseload midwifery, a one to one midwifery model of care in which women are cared for by a primary midwife throughout pregnancy, birth and the early postnatal period; a model of care that has been subjected to very little rigorous evaluation. We know of only two randomised controlled trials (RCTs) of caseload midwifery care; both conducted in the United Kingdom in the1990s [2,3]. One did *not *include an 'on call' component for midwives [2], whereby midwives are called in to work when a woman in their caseload requires labour care. This aspect is likely to have a significant impact on midwives' lives and has been a common component of the model when implemented in Australia. The other was a cluster trial, with all midwives attached to between one and three general medical practices [3] – a very different system of maternity care than that available in Australia. Other evaluations of the caseload model have used comparative descriptive designs, with most arguing that for feasibility and practical reasons using an RCT design was not possible [4-6]. There have been no RCTs of caseload midwifery care in Australia.

There is evidence from RCTs that continuity of midwifery care may lead to reduced caesarean sections [7,8] and instrumental vaginal births [9], and a decrease in other interventions during labour including induction [3,9] augmentation [9] analgesia use [9] and episiotomy [10,11]. One Australian RCT demonstrated a decrease in women having caesarean birth from 18% to 13% [7]. Many of these RCTs have also reported increased satisfaction for women [11-14], with no statistically significant differences in perinatal morbidity or mortality [9,15]. RCTs of continuity of midwifery care in the UK and in Australia have largely measured the effect of *teams *of care providers (commonly 6–12 midwives). Caseload midwifery care differs in that women are cared for by a *primary *midwife throughout pregnancy, birth and the early postnatal period. The underlying philosophy is one of continuity of carer for both women and midwives. The primary midwife is on call for labour and birth care for the women in her caseload. One or two other midwives are introduced during pregnancy in case they are needed as a back-up, for example if the primary midwife has two women in labour at the same time, if a woman's labour is quite extended or if the primary midwife is on days rostered 'off call' or leave when labour begins. A fulltime midwife usually cares for 40–45 women per year [16].

The impact of the caseload midwifery model on staff retention and attrition is unknown, but is another important issue for consideration in light of the fact that a 2002 review of the midwifery workforce in Australia concluded that there is a national shortage of midwives that is expected to increase over the next few years [17]. It is possible that the continuity inherent in caseload midwifery and potential for lower intervention childbirth would improve midwife satisfaction [18-21]; however studies in the UK and Australia have reported problems with the widespread implementation and organisation of models that promote continuity of carer. Issues for midwives include high and unsustainable workloads, personal costs (impinging on family life) [22,23]; and burnout and stress [24]. A caseload model in Sydney ceased operations in 2001 following "many stressors from within and beyond the partnership model" [[5], p34]. In response to these issues, some organisations have altered their approach, and moved from a caseload model to midwifery teams [25]. A 2000 review of continuity of carer models concluded that services should be organised in a way that aims to put less strain on midwives' lives [26]. Conflict between midwives working in new models and the staff in traditional models has also been reported [27,28]. Midwives in new models, or the new models themselves, may be seen as a threat by medical staff, in that the midwives may be taking on work otherwise done by them [21]. In a qualitative evaluation of a team midwifery model in Brisbane, midwives were surprised by the lack of support from other staff, both peers and administrators [27]. In two other trials it was reported that team midwives had to frequently respond to criticisms about their role or work practices [28]. In a setting of midwifery workforce shortages it is critical that the impact of new models of care is properly evaluated with regard to midwife job satisfaction, recruitment and retention.

There is a lack of evidence regarding the safety and the efficacy of the caseload model, although the existing RCTs of midwifery care (mostly team midwifery) do report decreases in interventions in labour and birth. In Victoria in 2004, 30% of births were by caesarean; a rate that has almost doubled over the past 20 years [29]. Reports in the USA and Australia have shown that the increase is related partly to non-clinical factors such as demographics, physician practice patterns, and maternal choice [30-34]. In Australia, intervention rates are highest among women with private health insurance, women giving birth in major tertiary hospitals and women attended by specialist obstetricians [34], and there is a particular concern with the high rate of elective caesarean section where there is no medical indication, and a recommendation that there should be national leadership to reduce caesarean section rates [34]. It is timely that an RCT be undertaken to test the safety of the caseload model and to ascertain the effect of caseload midwifery on the rate of caesarean section births.

There is increasing evidence that a caesarean section has implications for subsequent pregnancies, including increased risk of placenta accreta and percreta [35]; decreased fertility [36-40]; and an increased risk of ectopic pregnancy and spontaneous abortion [41]. Evidence around the protective effect (or otherwise) of caesarean section on urinary and faecal incontinence is inconsistent and likely to be multifactorial. There is some evidence of increased neonatal respiratory morbidity for babies born by caesarean section [42,43], however the frequency of significant fetal injury may be greater with vaginal delivery [44]. There are also significant resource implications: the increasing caesarean section rate adds an economic burden to already under-resourced medical systems [45].

Significant questions remain regarding the safety and efficacy of the caseload model, as well as its sustainability, the impact on the workforce, and costs of the model, given how different caseload is to a model using a team of midwives. There have been few economic evaluations of midwifery models of care. Published studies have reported conflicting results: some have reported continuity of care to be more expensive [46] and others that continuity models are more cost effective [14,47], although the studies used different methods to calculate costs.

It is not clear whether continuity of carer per se is more important to women than consistent and personalised care, even if it is provided by a number of care-givers [26,48].

We plan to implement a caseload midwifery model under RCT conditions to evaluate its effect on the rate of caesarean section and on a range of significant secondary outcomes. This paper describes the trial protocol in detail.

## Methods/design

The study uses a two arm, unblinded randomised controlled design, stratified by parity, to compare caseload midwifery care with standard maternity care.

### Aims

This study aims to determine whether caseload (one to one) midwifery care for women at low risk of medical complications decreases the proportion of women delivering by caesarean section compared with women receiving 'standard' care. The primary hypothesis is that:

Women randomised to caseload midwifery care will have 5% fewer caesarean section births than women in standard care model, that is, 14% versus 19%.

Secondary aims of the study are to compare caseload midwifery and standard care with regard to differences in:

a) Maternal outcomes

- instrumental vaginal births, obstetric analgesia, perineal trauma, and induction of labour;

- postnatal depression;

- satisfaction with care;

- breastfeeding duration;

- smoking cessation.

b) Midwife outcomes (compared to midwives providing standard care)

- staff attrition from the model of care and the hospital;

- work satisfaction;

- burnout.

c) Costs and cost effectiveness

A final aim is to collect data on maternal and perinatal morbidity and mortality for inclusion in meta-analyses (the sample size will be insufficient to determine statistical differences). These include measures of perinatal morbidity and mortality and maternal mortality and morbidity.

Comprehensive process evaluation will be undertaken to explore sustainability issues and to assess intervention compliance.

### Outcome variables

#### Primary outcome

The principal outcome of the study is the proportion of women having a caesarean section birth.

For the secondary aims of the study the following variables will be collected:

a) Instrumental vaginal births

b) Obstetric analgesia

c) Perineal trauma

d) Induction of labour and augmentation of labour

e) Satisfaction with care

f) Proportion of women breastfeeding to two and six months

g) Postnatal depression

h) Smoking

i) Costs

j) Staff attrition and satisfaction

#### Other related outcomes

measures of perinatal morbidity and mortality (e.g. fetal or neonatal death, intraventricular haemorrhage, necrotising enterocolitis, sepsis, severe respiratory distress syndrome, special care nursery admissions; Apgar scores, seizures); and maternal morbidity and mortality (e.g. postpartum haemorrhage, eclampsia, postpartum pyrexia, pulmonary embolus).

### Study population

Women attending for a booking visit at the antenatal clinic of a tertiary maternity hospital in Melbourne, Australia during the recruitment period and who are identified as being at low medical risk will be approached to participate in the trial.

#### Inclusion criteria

- English-speaking: able to speak, read and write in English;

- Less than 24 completed weeks gestation at recruitment;

- Low-medical risk at recruitment (list below);

- Singleton pregnancy.

#### Exclusion criteria

- Planned, elective caesarean section

- Considered to be at increased medical or obstetric risk according to the following criteria:

#### Medical criteria

○ Age > 42 years (subject to medical review)

○ Alcohol or drug abuse

○ History of anaesthetic difficulties (subject to medical review)

○ Anaemia (< 90 g/l not responding to treatment)

○ Asthma and/or chronic bronchitis (poorly controlled or requiring hospitalisation in the last 12 months)

○ Autoimmune diseases (includes antiphospholipid antibodies)

○ Bleeding disorders and/or haemolytic disease

○ Body mass index ≥ 35 or < 17

○ Cardiac disease

○ Diabetes mellitus

○ Endocrine disorders

○ Epilepsy requiring anticonvulsants in the previous twelve months

○ Gastrointestinal disorders (subject to medical review)

○ Haemoglobinopathy (major)

○ HIV positive status

○ Hypertension

○ Infectious disease (e.g. Acute Hepatitis C)

○ Neurological disorders

○ Severe psychiatric illness (subject to psychiatric advice)

○ Physical disability (e.g. paraplegia)

○ Renal disease

○ Respiratory disease (chronic/disabling)

○ Thromboembolic disease

○ Thyroid disease (uncontrolled)

#### Obstetric/gynaecological criteria

○ Birthweight of previous baby < 2500 g or > 4500 g (subject to medical review)

○ Significant cervical conditions (e.g. previous cone biopsy)

○ Fetal abnormality requiring specialised neonatal care

○ Previous mid trimester loss (× 2)

○ Multiple pregnancy

○ Parity ≥ 5 (subject to medical review)

○ Placental abruption (previous significant)

○ Previous significant postpartum haemorrhage (subject to medical review)

○ Pre-eclampsia (previous)

○ Puerperal psychosis (previous)

○ Recurrent miscarriage (× 3)

○ Rhesus isoimmunisation or other significant blood group antibodies

○ Previous stillbirth (not due to congenital malformation)

○ Uterine surgery (e.g. previous caesarean section)

○ Uterine myoma or malformation (subject to medical review)

### Sample size

Initial power calculations were based on the caesarean section rate for women who were low risk at booking at the Royal Women's hospital in 2005, i.e. 19%. It was hypothesised that the caseload model of care would decrease the caesarean section rate from 19% to 14%. In order to detect such a difference (with 80% power and 95% confidence) we needed 904 women in each trial arm. Allowing for 10% loss to follow up, 1004 women in each group were required, i.e. a total sample of 2008. This sample size also enables the detection of a range of other statistically significant differences to be detected for the major secondary outcomes, as shown in Table 1.

**Table 1:** Power calculations with n = 904** in each arm

*Outcome*	*Standard care*	*Caseload*	*Power to detect specified difference*
	%	%	
Birth by caesarean section	19	14	80
Instrumental vaginal birth	10.6	6.6	84
Epidural analgesia	18	13	82
Intact perineum or unsutured laceration (increase)*	41.2	48.2	84
Intact perineum or unsutured laceration (decrease)*	41.2	34.2	86
3rd or 4th degree laceration* (would not miss this difference)	1.7	3.4	57
Induction of labour	27	21	84
Augmentation of labour	19	14	80
Exclusive breastfeeding at 6 months	8	12	79
Any breastfeeding at 6 months	53	63	99
Postnatal depression at 8 weeks	15	10	88
Postnatal depression at 6 months	15	10	88
Smoking at time of birth	15	10	88

*These estimations exclude caesarean births

** Allows for loss to follow-up

### Recruitment of women to the trial

Recruitment to the trial will take place in the antenatal clinic when women attend for their maternity booking visit.

#### Participant information

Women will be sent written information (a brochure) about the study when they ring to book into the hospital for their pregnancy care (see Additional file 1). A participant information sheet and another copy of the brochure will be given to each woman at the time of recruitment. Women will be given the opportunity to read the information sheet, think about the study, and then consent if they wish.

#### Informed consent

Written consent will be obtained if a woman agrees to enter the study. This will be witnessed and signed by another person, as well as the research midwife.

#### Assessment of eligibility

Research midwives will liaise with midwifery and clerical staff in the antenatal clinics to obtain daily booking visit lists. A preliminary assessment of a woman's study eligibility will be made by checking the woman's medical record.

#### Recruitment protocol

Women will be approached prior to or immediately after completion of the midwife booking visit. Research midwives will follow a recruitment guide after determining that a woman is potentially eligible.

If a woman does not wish to decide immediately, she will be provided with study contact details, a copy of the consent form and background questionnaire, and a reply paid envelope. To join the study she can then contact the research midwife and return the forms by mail. Upon the return of the forms, the woman will be randomised and contacted regarding the outcome of the random allocation.

### Intervention allocation

#### Randomisation procedure

The randomisation system in use for the trial was developed by the Australian National Health and Medical Research Centre Clinical Trials Centre . An Interactive Voice Response System (IVRS) activated by telephone will be used for randomising women to the two treatment arms of the trial (i.e. standard care and caseload midwifery care).

Randomisation will be undertaken using stratified permuted blocks of varying size, with randomisation stratified by parity (i.e. first or subsequent birth). For each woman, the system will assign the treatment in the next available sequential slot in the appropriate list of blocks.

When a woman agrees to enter the study the research midwife will complete a COSMOS study specific Trial Randomisation IVRS Worksheet with details that the system will request to complete the randomisation procedure (e.g. woman's full initials, parity and date of birth). Access to the IVRS is achieved by dialing a toll-free telephone number and navigating to the COSMOS trial via a menu system. The system is designed so that no woman can be randomised twice. A back-up system is in place in case of a system failure, via a nominated person at the Clinical Trial Centre. After completing all prompts the woman will be allocated a study number and treatment arm. The woman will be informed immediately of the randomisation outcome and further maternity care bookings arranged accordingly.

### Study participation

#### Baseline

After consenting to participate in the study and prior to randomisation, women will be asked to complete a baseline questionnaire, which includes demographic data.

#### Intervention

Women will be allocated to either the control or intervention arms (described below).

#### Follow-up

Further data collection is scheduled to take place following the birth (obstetric and medical data obtained from hospital record) and self-completed participant questionnaires at two and six months postpartum (postal). The hospital records will be checked following the birth before sending postal questionnaires to minimise the chance of a woman who has experienced a fetal death or an early neonatal death receiving a questionnaire. Women who are seriously ill or who have had a seriously ill infant will be sent questionnaires subject to confirmation that they have been discharged from hospital in good health. Where there are any concerns regarding whether a woman should be sent a questionnaire, the research team will discuss the issue and reach agreement.

#### Study completion

Successful completion of the study occurs after the six-month questionnaire is returned. If desired, women will be sent a summary of the results after completion of the analyses.

#### Participant discontinuation

Women are free to withdraw from the study at any time. No further study follow-up will occur.

### Interventions

#### Description of caseload care

Women allocated to the caseload intervention will receive antenatal, intrapartum and postpartum care from a primary caseload midwife with one or two antenatal visits to be conducted by a 'back-up' midwife. The primary midwife will be on call for the woman's labour and birth except in designated circumstances such as annual leave; sick leave; having already worked more than 12 hours in a 24 hour period; having more than one woman in labour; or if it is on one of the two days per week that the primary midwife is scheduled to not work or be on call. Care will then be provided by a back-up midwife or on occasion by non-caseload midwives. Negotiation of these details will be organised within each caseload group, as autonomy and control over working times and conditions have been shown to be important aspects of preventing burnout [24]. The primary midwife will collaborate with obstetricians and other health professionals as necessary, and will continue to provide caseload care in addition to care provided by obstetricians if complications develop. In addition to providing care until after the birth of the baby, the primary midwife (or a back up midwife) will attend the hospital on most days to provide some postnatal care and will provide domiciliary care following discharge from hospital. Care will be provided according to hospital guidelines and protocols.

#### Recruitment of midwives to caseload

Approximately 12 fulltime midwives are required to staff the intervention. This can consist of more actual midwives (e.g. it could be several part time midwives). Positions will be advertised in the first instance internally, then if the required number is not met there will be external advertisements. To be eligible, midwives need to have completed two years clinical midwifery post-registration.

#### Education of caseload midwives

In addition to any education deemed to be necessary by the hospital in preparation for working in the caseload model, caseload midwives will be offered a one day training session by the research team. One third of the day will focus on the research components of the trial and the rest will be in regard to working in this new way-practicalities, personal boundaries, and working in a small group (see Additional file 2 for program outline).

#### Description of standard care

Women allocated to the control group can choose from the standard hospital options for low risk women; these include midwifery-led care – which may involve some continuity or could mean women seeing a different midwife for every visit; care by junior medical obstetric staff; or community based care – usually shared care with an accredited general medical practitioner (GP) (i.e. the GP provides the majority of the woman's antenatal care, usually nearer to her home, but the woman is booked for labour, birth and postnatal care at the hospital). In both the midwife-led and GP-led models women would be booked to see an obstetrician at 36 and 42 weeks gestation, with other referral or consultation as necessary. When women come into the hospital for labour, birth and postnatal care they will be cared for by whichever midwives and doctors are rostered for duty. Care will be provided according to the same hospital guidelines and protocols as for the women in the intervention arm.

It is possible that some women allocated to either the intervention or the control group will seek other care options, such as care with a private obstetrician or midwife, but neither of these is available at the Royal Women's Hospital. In this event we aim to obtain all data as planned, and analysis will be by intention-to-treat.

### Process evaluation

Adherence to the study protocol will be monitored and intervention fidelity measured in a range of ways.

#### Measures of intervention exposure

Data regarding the extent to which care was provided by the primary midwife will be collected from the medical record following the birth and from the women via postal questionnaire at two months. To assess and compare continuity of carer, women in both trial arms will be asked about the presence of known care providers for labour, birth, postnatal hospital care and domiciliary care.

#### Caseload midwife meetings

Monthly team meetings will be held with the caseload midwives and a member of the research team (DF) to provide support and give an opportunity for problem-solving and debriefing. The research team member will be available to the caseload midwives before and after meetings and will be contactable between meetings by email or telephone. Protocol adherence will be discussed at team meetings. Other key support for the caseload midwives will be from their clinical manager(s) as well as from the Director of Maternity Services (TF).

#### Adherence to protocols

Three sources will contribute to assessing adherence to the intervention protocols: interviews with the intervention midwives at the beginning and end of the trial; monthly meetings between the caseload midwives and the research team member; and data collection from the medical records.

#### Intervention evaluation by participants

The postal questionnaire sent to women at two months will include a number of questions about women's model of care and continuity of carer. Questions will also assess satisfaction with model of care provision.

#### Intervention evaluation by caseload midwives, non-caseload midwives and other key stakeholders

Comprehensive evaluation of how the intervention works within the organisation will be undertaken using surveys and key informant interviews.

### Data collection

#### Trial participants

○ A background demographic data questionnaire will be completed by women at the time of recruitment, prior to randomisation.

○ Obstetric/medical data will be collected manually from the medical record following the birth.

○ Data will be collected at two and six months postpartum by postal survey. Questionnaires will be mailed by the research midwives and/or project coordinator. A covering letter will be sent with each questionnaire and a reply paid envelope included. To maximise response, a reminder letter will be sent two weeks after the first mailing, then if there is no response in another two weeks women will receive a second reminder letter that includes another copy of the questionnaire. When these questionnaires are returned they will be checked for women's responses to the self harm question (Q10) on the Edinburgh Postnatal Depression Scale [49], and if a woman has indicated that the thought about harming herself has occurred to her "quite often" in the last week she will be telephoned according to a pre-specified protocol (see Additional file 3).

#### Caseload midwives, non-caseload midwives and other key stakeholders

○ *A *questionnaire will be administered to all caseload midwives and non-caseload midwives near the beginning and at completion of the trial. In-depth interviews will also be conducted with all caseload midwives and a sample of non-caseload midwives and other key stakeholders, near the beginning of the trial and on completion. These will be audio-taped then transcribed verbatim.

○ Midwife retention rates for caseload midwives and non-caseload midwives will be estimated at baseline and post-trial to enable a comparison.

○ Logs of caseload midwives' time spent on their various work tasks will be collected for several one-month intervals throughout the trial.

#### Economic evaluation

○ Resource use data will be collected from the medical record following birth and from women's self-reported use of health care and other resources in the six months after birth, via the postal questionnaires at two and six months postpartum.

### Data coding

Data coding schedules have been devised for each survey questionnaire, and coding will be undertaken as the questionnaires are returned.

### Data management

#### Data storage

Data will be stored in locked filing cabinets in locked rooms and accessed only by the project coordinator and chief investigators. Computer files will be password protected and will be accessed only by the project coordinator and chief investigators. Data linking women's names and study ID will be kept on a separate database to women's questionnaires which will be identified by study ID only.

#### Data entry

Survey data will be entered by a professional data entry company then the files exported to an Access database [50].

Qualitative data entry: open-ended questions from the surveys will be entered by the project coordinator. Key informant interviews will be transcribed verbatim by an external professional transcriber. Analysis will be undertaken by members of the research team.

#### Data cleaning

Data cleaning for quantitative data will be undertaken using a variety of approaches including range and logical checks, first in Access [50] then again when the data is transferred to the statistical software package STATA [51].

### Data analysis

#### Quantitative analysis

This will be undertaken using STATA [51] and data will be collected to meet the CONSORT guidelines for reporting of randomised trials.

The first stage of analysis will check the comparability of participants allocated to the two groups. The intervention group will be compared with the standard care group by intention to treat analysis. Proportions of women having a caesarean section will be compared using chi-square tests and odds/risk ratios. Comparison of means will be undertaken for continuous variables using t-tests where data are normally distributed or medians compared using Mann-Whitney U tests otherwise. Ranked or Likert-type scales will be analysed using cumulative odds ratios. Where there are differences in baseline characteristics of the women in the two groups which might be associated with outcomes, an additional multivariate analysis will be carried out.

#### Qualitative analysis

Analysis of open-ended questions from surveys will be undertaken using simple thematic analysis and coded into themes. Key informant interviews will be transcribed verbatim and the transcripts checked against the audiotape for accuracy. A thematic network will be constructed using electronic and paper copies of the transcripts from the interviews, as a way of organising the thematic analysis, providing emerging basic, organising, and global themes to describe the data [52]. Transcripts will be read and reread to gain an overall perspective then a step by step approach used. A coding framework will be developed to reduce the text to meaningful manageable parts. Basic emerging themes will be identified, then summarised into more abstract groups (organising themes) and finally summarised as overriding metaphors, or global themes [52].

### Study administration

#### Personnel

Principal investigators

Dr. Helen McLachlan (Mother and Child Health Research & Division of Nursing & Midwifery, La Trobe University)

Dr. Della Forster (Mother and Child Health Research, La Trobe University & Royal Women's Hospital)

Ms. Mary-Ann Davey (Mother and Child Health Research, La Trobe University & Consultative Council on Obstetric and Paediatric Mortality and Morbidity (CCOPMM), Victorian Department of Human Services)

Professor Judith Lumley (Mother and Child Health Research, La Trobe University)

Ms Tanya Farrell (Royal Women's Hospital)

Professor Jeremy Oats (initially Royal Women's Hospital, later CCOPMM, Victorian Department of Human Services)

Ms. Lisa Gold (Deakin University)

Professor Ulla Waldenström (Karolinska Institutet, Stockholm)

#### Associate investigators

Dr. Mary Anne Biro (Mother and Child Health Research, La Trobe University)

Professor Leah Albers (University of New Mexico, USA)

#### Project team

Helen McLachlan

Della Forster

Mary-Ann Davey

Judith Lumley

Tanya Farrell

Mary Anne Biro

Lisa Gold

Michelle Newton

#### Project coordinator

Mary Anne Biro

#### Research midwives

Michelle Kealy

Sue Veljanovski

Jo Rayner

Cath Fitzsimon

#### Health economics research assistant

Bree Rankin

#### Job descriptions of required personnel

Position descriptions: Project coordinator; Research Midwife and Health economics research assistant (see Additional file 4)

### Staff education

Research midwives will be required to undertake one day of initial orientation regarding the project overall, including the data collection tools, the recruitment process and interview techniques. This process will continue with supervised recruitment, and then continued close contact by the project coordinator.

### Staff meetings

It is anticipated that the research project team will meet monthly throughout the project, and more often as necessary.

### Study reference group

A reference group will be established to bring together a group of people from a range of backgrounds, with relevant expertise and interest in the trial. They will contribute ideas and advice to the research team through all stages of the project; comment on drafts of materials and resources developed to support the project (e.g. questionnaires); provide advice and assistance in promoting the project where appropriate; participate in discussion of the findings and their implications for health service delivery across Victoria and assist in developing appropriate strategies for disseminating the findings of the project. Terms of reference have been developed (see Additional file 5). Responsibility for conduct of the study remains with the principal investigators.

### Timelines

This trial is expected to take four years which includes recruitment of 85 women per month, requiring 24 months for recruitment. Medical data will be collected shortly after the birth, then a postal questionnaire will be mailed to women at two and six months postpartum. Following recruitment of the final woman to the study, it will take 14 months to complete data collection. The fourth year will be required to finalise data collection and analysis.

### Documents required

#### Information brochure

An information brochure will be sent to all women when they ring and book into the hospital and will also be given to women at recruitment (see Additional file 1).

#### Participant information

Eligible women who are approached to participate in the study will be given a participant information sheet.

#### Consent form

Written consent will be obtained if the woman agrees to enter the study. This will be witnessed by another person as well as the research midwife.

#### Data collection forms/software

- Background demographic questionnaire

- Two month questionnaire

- Six month questionnaire

- Medical/obstetric outcome collection form

- Daily recruitment figures form

- Excel workbook to track recruitment and record data related to population numbers, proportion approached and consented, and reasons for non-participation [53]

- Access database to track participants [50]

- Staff surveys at start and end of trial

#### Coding schedules

Coding schedules for each data collection form.

### Ethical considerations

#### Ethics approvals

Approvals were received from

Research and Ethics Committees, Royal Women's Hospital (No. 07/01)

University Human Ethics Committee, La Trobe University (No. 07-04)

#### Informed written consent

Written consent will be obtained if a woman agrees to enter the study. This will be witnessed by another person, as well as the research midwife. Written consent will also be obtained from all staff members who are offered and agree to an in-depth interview and from all caseload midwives who agree to participate in the various aspects of data collection pertaining to them.

#### Risks/inconvenience/benefits

We do not anticipate any potential or actual harms of participation. Many RCTs of continuity of midwifery care compared with standard medical care have reported no statistically significant differences in perinatal morbidity or mortality and have reported increased satisfaction with care in the continuity of midwifery arm. The model to be trialed focuses much more on continuity of carer rather than continuity by a team of carers, and thus benefits or potential harms will not be known until results are available.

This study includes women who are at low medical risk and excludes women at high risk. A strict protocol will be followed for women who develop complications. If a woman in the intervention arm develops medical or obstetric complications she will be transferred to medical care as per the hospital protocol, but in addition will continue to receive caseload midwifery care.

There is evidence from RCTs that continuity of midwifery care may lead to reduced caesarean sections and operative vaginal births, and a decrease in other interventions in labour including induction, augmentation, analgesia use and episiotomy.

### Data monitoring and safety committees

A data monitoring committee will be established comprising an external statistician, a clinical expert and an expert in RCTs to check the randomisation and undertake an interim analysis. A safety committee, including members with relevant expertise, who are blinded to group allocation, will be established to review reports of any adverse events. Additional file 5 includes the terms of reference for these committees.

#### Interim analysis

The data monitoring committee will conduct an interim analysis on the medical/obstetric outcome data and recruitment rate when 500 births have occurred in each arm of the trial.

## Abbreviations

COSMOS: COmparing Standard Maternity care with One to one midwifery Support; GP: General medical practitioner; NHMRC: National Health and Medical Research Council; RCT: Randomised controlled trial; RWH: Royal Women's Hospital (Melbourne).

## Competing interests

The authors declare that they have no competing interests.

## Authors' contributions

HM, DF, MAD are chief investigators and have joint overall responsibility for the trial; HM, DF, MAD, TF, LG originally conceived the study; HM, DF, MAD, JL, UW, LG, JO, LA designed the study; HM, DF, MAD, TF, LG, LA wrote the initial grant application; HM, DF, MAD, LG drafted the initial study protocol; HM, DF, MAD, LG drafted the first questionnaire drafts; HM, DF, MAD, MAB, LG undertook questionnaire development, piloting and completion; HM, DF, MAD, MAB, LG, JL, JO, LA reviewed the questionnaires; HM, DF, MAD, TF, JO conceived the intervention; DF, TF, MAB developed the intervention; DF, TF responsible for implementation of the intervention MAB, DF, TF, HM, MAD implemented and co-ordinated the trial; HM, DF, MAD, MAB developed the process evaluation measures; HM, DF, MAD, MAB, LG, JL responsible for ongoing review of trial processes; HM, DF, MAD, MAB, LG developed data collection processes; MAB, HM, DF, MAD, LG data collection and management responsibility; HM, DF, MAD, LG, MAB will undertake data analysis; and HM, DF, MAD, MAB drafted the trial protocol manuscript. All authors read and approved the final manuscript.

## Funding

Funding has been obtained from the Australian National Health and Medical Research Council (Project Grant 433040).

## Pre-publication history

The pre-publication history for this paper can be accessed here:



## Supplementary Material

Additional file 1 Information brochure. Click here for file

Additional file 2 Orientation and training program outline for intervention midwives. Click here for file

Additional file 3 Protocol for responding to women who may be depressed. Click here for file

Additional file 4 Position descriptions. Click here for file

Additional file 5 Terms of Reference for Reference group; Safety Committee and Data Monitoring Committee. Click here for file
