# Supplementary material for: COSMOS: COmparing Standard Maternity care with One-to-one midwifery Support: a randomised controlled trial
Source: BMC Pregnancy Childbirth. 2008 Aug 5;8:35. doi: 10.1186/1471-2393-8-35 (PMC2526977; doi:10.1186/1471-2393-8-35)
Supplement: Additional file 1 — Information brochure. [file 1471-2393-8-35-S1.pdf]

how do I join?

Just tell the midwife at your booking visit if you are interested. You will then meet a member of the research team who will give you more information about the project and answer any questions you have.

cosmos

more information

Please call the *'One to one midwifery'* Project Team on 8341 8536

COMPARING STANDARD MATERNITY CARE WITH 'ONE TO ONE' MIDWIFERY SUPPORT

(COSMOS)

the women's
the royal women's hospital
victoria australia

mother & child
HEALTH RESEARCH

LA TROBE UNIVERSITY

RESEARCHING A NEW FORM OF CARE

The Women's is planning to introduce a model of maternity care called '*one to one midwifery*'. One to one midwifery care is a system of care that involves you being cared for by the same midwife during pregnancy, labour, birth and after the birth.

We are evaluating this new form of care and comparing it with our existing models of maternity care.

the research project

'*One to one midwifery* care' has been introduced in other places and is now being introduced at the Women's for the first time. We consider it important to evaluate any new systems of care, so we want to compare it with the models of maternity care currently available at the Women's.

If you are interested in *one to one midwifery* care and agree to join the research project you will be allocated randomly (by chance) to receiving *one to one midwifery* care or to receiving usual care. That is, half of the women interested in this project will receive the new model of care and half will receive one of the existing models. If you are allocated to the usual care group you will be able to choose from all the types of care normally available at the Women's.

After the birth we will ask you to fill out two questionnaires about the care you received - one six weeks after the birth, and one six months after the birth. The questionnaire will be returned to the research team. With your permission we will collect some information about your pregnancy and birth from your hospital record. The information you provide will be extremely valuable in helping us plan our future services. All information will be treated as completely confidential.

how does '*one to one midwifery* care' work?

One to one midwifery is a system of care that involves you being cared for by a midwife who will undertake most of your check-ups during pregnancy with one or two visits conducted by a 'back-up' midwife. Your midwife will normally be there to care for you during your labour and birth, and will provide some care for you and your baby after the birth. Your midwife may not be available if she is off duty, but if she is not able to be with you, one of the other midwives you have seen during pregnancy will usually be available to care for you during labour and birth. Should any medical complications occur during pregnancy or labour, care will also be provided by a doctor.

am I eligible to participate in this project?

Anyone at low risk of medical complications, who is able to read and write English and is less than 24 weeks pregnant and who is interested in participating in the research project is likely to be eligible.
