# Supplementary material for: COSMOS: COmparing Standard Maternity care with One-to-one midwifery Support: a randomised controlled trial
Source: BMC Pregnancy Childbirth. 2008 Aug 5;8:35. doi: 10.1186/1471-2393-8-35 (PMC2526977; doi:10.1186/1471-2393-8-35)
Supplement: Additional file 2 — Orientation and training program outline for intervention midwives. [file 1471-2393-8-35-S2.doc]

**Additional file II: Orientation/training program for one to one midwifery (COSMOS)**

**Orientation session for one to one midwives**

**Where: Mother & Child Health Research, 324-328 Little Lonsdale Street, Melbourne (located between Elizabeth and Queen St)**

**Date: Wednesday 14^th^ November 2007**

**Time: 0830-1700**

**Objectives**

- - To introduce the COSMOS trial, RCT methodology and working as a one to one midwife in a research setting
  - To discuss support processes for one to one midwives
  - To explore the differences between one to one midwifery and current practice
  - To explore the meaning of working in a one to one practice
  - To explore the practicalities of working in a one to one practice
  - To provide strategies for developing effective working relationships with
    - women
    - one to one colleagues
    - core midwifery colleagues
    - medical colleagues
  - To provide strategies for dealing with organisational change

**8.15-8.30am** Registration/Coffee

**8.30-8.50am Personal introductions (all)**

- - - Research team
    - One to one midwives
    - Recruitment midwives

**8.50-9.30am Researching one to one midwifery care**

- - - - Implementing the model
      - Researching the model
      - What is a randomised trial? Why this methodology? Why the research at all?
      - What are we measuring?
      - Economic evaluation
      - What does it mean to be part of the intervention?
      - What are the one to one midwife’s responsibilities in the trial?
      - What are the research midwife’s responsibilities?

**9.30-9.45am Morning tea**

**9.45am-12pm Working in one to one midwifery**

- - - What is different between one to one and current midwifery practice-midwives’ brainstorming exercise
    - What’s it like to work in one to one midwifery practice?
    - The practicalities
      - Keeping a diary
      - Reconciling hours and payment
      - Managing the workload
      - Time management
      - Setting priorities
      - Managing/sharing on-call working arrangements-coping with the pager/phone
    - Balancing professional and personal lives
    - Working with a buddy or two
    - Reporting structures
    - Taking accountability
    - Knowing the limits-working within guidelines and protocols
    - Clinical decision-making within one to one
    - What does the job description actually require of me?
    - What other clinical skills do I need to be a one to one midwife? Aneeds assessment

**12-12.45pm Lunch**

**12.45-4.45pm Establishing and maintaining positive relationships**

- - - Working with women in a continuity of carer relationship
      - Practicing? in a caring professional relationship
      - Avoiding dependency
    - Developing effective working relationships with
      - Other one to one midwives
      - Core/unit midwives
      - Medical staff
    - What’s good communication?
    - What is the difference between interdependence and independence?
    - How do I cope with change?
    - How do I deal with difficult behaviour?
    - Who do I go to for support?
    - Role-modelling of potential scenarios

**4.45-5pm Summing up**
