# Supplementary material for: COSMOS: COmparing Standard Maternity care with One-to-one midwifery Support: a randomised controlled trial
Source: BMC Pregnancy Childbirth. 2008 Aug 5;8:35. doi: 10.1186/1471-2393-8-35 (PMC2526977; doi:10.1186/1471-2393-8-35)
Supplement: Additional file 3 — Protocol for responding to women who may be depressed. [file 1471-2393-8-35-S3.doc]

**Additional file III: Protocol for responding to women who may be depressed**

Postnatal depression will be measured using the “Edinburgh postnatal depression scale” (EPDS) which will be included in the 2 and 6 month postal questionnaire. The EPDS includes a question “During the last week the thought of harming myself has occurred to me”, with response alternatives including: Yes, quite often; sometimes; hardly ever or never.

This item attempts to identify women who are considering self-harm or suicide. However experience in the use of this instrument has shown that women sometimes respond positively to this question without any thoughts of self harm because of a misunderstanding; or the delay between completing the item and receipt of the questionnaire by the study team enables a temporarily distressing situation to improve.

We will monitor the response to this item as soon as the questionnaire is received. If we receive a questionnaire with the response “Yes, quite often or sometimes”, the project co-ordinator (or Chief Investigator) will attempt to contact the participant by telephone. The project coordinator will follow a protocol which will include:

- Explain the reason for the call and explore the intention of the response. If the woman has thought of harming herself, discuss some possible avenues for obtaining further support in relation to her individual situation.
- Explore whether she knows anyone to talk to, and encourage her to talk to them if she feels they will understand and be supportive.
- Discuss options for obtaining extra support, such as organising time for herself away from the baby, contacting support groups for new mothers, or neighbourhood houses.
- Offer to facilitate putting the woman in contact with someone able to provide support (GP, maternal and child health nurse, community health centre, mother and baby units).
