# Supplementary material for: COSMOS: COmparing Standard Maternity care with One-to-one midwifery Support: a randomised controlled trial
Source: BMC Pregnancy Childbirth. 2008 Aug 5;8:35. doi: 10.1186/1471-2393-8-35 (PMC2526977; doi:10.1186/1471-2393-8-35)
Supplement: Additional file 4 — Position descriptions. [file 1471-2393-8-35-S4.doc]

**Additional file IV: Position descriptions for COSMOS trial including research assistant: health economics; research midwife; project co-ordinator**

# POSITION DESCRIPTION: HEALTH ECONOMICS RESEARCH ASSISTANT

### POSITION SUMMARY

| Mother and Child Health Research (MCHR) is a multidisciplinary research centre in the Faculty of Health Sciences at La Trobe University, which aims to:   - undertake and interpret research on mothers' and children's health; - contribute to policy development; - participate in postgraduate and continuing education; and - provide advice and resources to researchers in related fields.   The primary research focus of the Centre is in the areas of: health services, pregnancy and birth; perinatal and child health outcomes; cross-cultural and Indigenous issues and longer term health outcomes of reproduction and assisted conception. The criteria for choosing research topics are that they are major public health issues in terms of the burden of disease, the implications for women and their families, and/or the resource implications of the condition for health services or for society as a whole.  MCHR has a strong interest in health services research and is now building on observational studies to design and implement intervention studies in hospitals, primary care and community settings. Our evaluation methods include health outcome assessment, and process and impact measures, making use of both quantitative and qualitative methods.  A major focus of a number of MCHR projects is the maternal sequelae of reproductive events, in particular antecedents of preterm birth and physical and psychological disorders initiated or aggravated by pregnancy, labour or birth and intimate partner violence. Studies addressing these issues include use of routinely collected data and record linkage, observational surveys and interview studies, cohort studies and randomised trials with long term follow-up of participants. Another focus of MCHR work is the health and childbearing experiences of Indigenous and immigrant and refugee women, and the development of culturally relevant research methods and approaches.  **The Project:**  The COSMOS project is a multi-centre randomised controlled trial, comparing caseload midwifery care with standard maternity care.  The project involves   - Recruitment of 2008 women randomised to caseload midwifery care or standard maternity care. - Collection of baseline and outcome data for all women via questionnaires and medical record abstraction - Collection of data related to the implementation of the project and the experiences of staff. - Economic evaluation   **Primary Objective**  The position of Research Assistant has been established to undertake to assist with the health economic evaluation of the trial. |
| --- |

### DUTIES AND RESPONSIBILITIES

| 1. | Pilot economic data collection tools | 5% |
| --- | --- | --- |
| 2. | Economic data collection from hospital records | 70% |
| 3. | Conduct preliminary economic analyses  (under direction and supervision of health economist ) | 10% |
| 4. | Liaison with hospital finance departments and midwifery and medical staff at study sites as required | 5% |
| 5. | Undertake other Research Assistant duties as necessary | 10% |

### SELECTION CRITERIA

|  | Essential Requirements |
| --- | --- |
| 1. | Experience in the design, conduct and analysis of economic data |
| 2. | Excellent written and verbal communication skills |
| 3. | Ability to work in a multidisciplinary research team |
|  | Desirable Attributes |
| 1. | Experience in economic evaluation in randomised controlled trials |

# POSITION DESCRIPTION: RESEARCH MIDWIFE

### POSITION SUMMARY

| **Background:**  Mother and Child Health Research (MCHR) is a multidisciplinary research centre in the Faculty of Health Sciences at La Trobe University, which aims to:   - undertake and interpret research on mothers' and children's health; - contribute to policy development; - participate in postgraduate and continuing education; and - provide advice and resources to researchers in related fields.   The primary research focus of the Centre is in the areas of: health services, pregnancy and birth; perinatal and child health outcomes; cross-cultural and Indigenous issues and longer term health outcomes of reproduction and assisted conception. The criteria for choosing research topics are that they are major public health issues in terms of the burden of disease, the implications for women and their families, and/or the resource implications of the condition for health services or for society as a whole.  MCHR has a strong interest in health services research and is now building on observational studies to design and implement intervention studies in hospitals, primary care and community settings. Our evaluation methods include health outcome assessment, and process and impact measures, making use of both quantitative and qualitative methods.  A major focus of a number of MCHR projects is the maternal sequelae of reproductive events, in particular antecedents of preterm birth and physical and psychological disorders initiated or aggravated by pregnancy, labour or birth and intimate partner violence. Studies addressing these issues include use of routinely collected data and record linkage, observational surveys and interview studies, cohort studies and randomised trials with long term follow-up of participants. Another focus of MCHR work is the health and childbearing experiences of Indigenous and immigrant and refugee women, and the development of culturally relevant research methods and approaches.  **The Project:**  The COSMOS project is a randomised controlled trial, comparing caseload midwifery care with standard maternity care.  The project involves   - Recruitment of 2008 women to be randomised to caseload midwifery care or standard maternity care. - Orientation and on-going support for midwives working in the caseload model of care - Collection of baseline and outcome data for all women via questionnaires and medical record abstraction - Collection of data related to the implementation of the project and the experiences of staff.   **Primary Objective**  This position has been established to assist the project coordinator in managing the day to day running of the COSMOS trial |
| --- |

### DUTIES AND RESPONSIBILITIES

| Under the supervision of the project coordinator, and working closely with the research team and hospital personnel, the occupant of this position will: | | |
| --- | --- | --- |
| 1. | Recruit women to the trial using the COSMOS recruitment protocol. | 80% |
| 2. | Collect data from medical records using COSMOS specific data collection tools | 10% |
| 3. | Enter data onto a computer database | 5% |
| 4. | Undertake other duties as determined by the project coordinator | 5% |

### SELECTION CRITERIA

|  | Essential Requirements |
| --- | --- |
| 1. | Excellent written and verbal communication skills |
| 2. | Excellent organisational skills and attention to detail |
| 3. | Ability to maintain accurate records in a timely manner |
| 4. | Basic computing skills in word processing and data entry |
| 5. | Ability to work effectively in a multi-disciplinary research team |
| 6. | Nursing and/or midwifery qualifications |
|  | Desirable Attributes |
| 1. | Previous research experience preferably with randomised controlled trials |
| 2. | Previous experience with recruitment to a randomised controlled trial |
| 3. | Previous experience with data management |
| 4. | Hospital-based research experience and/or knowledge of the public maternity sector |

# POSITION DESCRIPTION: PROJECT COORDINATOR COSMOS

# POSITION SUMMARY

| Mother and Child Health Research (MCHR) is a multidisciplinary research centre in the Faculty of Health Sciences at La Trobe University, which aims to:   - undertake and interpret research on mothers' and children's health; - contribute to policy development; - participate in postgraduate and continuing education; and - provide advice and resources to researchers in related fields.   The primary research focus of the Centre is in the areas of: health services, pregnancy and birth; perinatal and child health outcomes; cross-cultural and Indigenous issues and longer term health outcomes of reproduction and assisted conception. The criteria for choosing research topics are that they are major public health issues in terms of the burden of disease, the implications for women and their families, and/or the resource implications of the condition for health services or for society as a whole.  MCHR has a strong interest in health services research and is now building on observational studies to design and implement intervention studies in hospitals, primary care and community settings. Our evaluation methods include health outcome assessment, and process and impact measures, making use of both quantitative and qualitative methods.  A major focus of a number of MCHR projects is the maternal sequelae of reproductive events, in particular antecedents of preterm birth and physical and psychological disorders initiated or aggravated by pregnancy, labour or birth and intimate partner violence. Studies addressing these issues include use of routinely collected data and record linkage, observational surveys and interview studies, cohort studies and randomised trials with long term follow-up of participants. Another focus of MCHR work is the health and childbearing experiences of Indigenous and immigrant and refugee women, and the development of culturally relevant research methods and approaches.  **The Project:**  The COSMOS project is a randomised controlled trial, comparing caseload midwifery care with standard maternity care.  The project involves   - Recruitment of 2008 women willing to be randomised to caseload midwifery care or standard maternity care. - Provision of caseload midwifery care to women so randomised. - Orientation and on-going support for midwives working in the caseload model of care - Collection of baseline and outcome data for all women via questionnaires and medical record abstraction - Collection of data related to the implementation of the project and the experiences of staff.   **Primary Objectives:**  This position has been established to have day-to day responsibility for the implementation and conduct of the COSMOS project. Overall trial responsibility remains with CIs A,B,C |
| --- |

### DUTIES AND RESPONSIBILITIES

| The occupant of this position must be able to Co-ordinate the COSMOS trial. This includes working as part of the COSMOS research team and engaging in regular team meetings, and specifically to: | | |
| --- | --- | --- |
| 1. | Manage trial on day to day basis e.g. organise team meetings, coordinate files, liaise with team, manage issues that require immediate resolution. | 10% |
| 2. | Oversee and participate in recruitment of women to the trial | 20% |
| 3. | Oversee randomisation procedures, process evaluation measures and adherence to protocols | 10% |
| 4. | Support intervention midwives eg via team meetings | 10% |
| 5. | Manage and support research midwives | 10% |
| 6. | Participate in design and piloting of questionnaires and databases | 15% |
| 7. | Liaise with hospital staff and other investigators | 5% |
| 8. | Monitor progress of the trial | 5% |
| 9. | Ongoing management of ethics processes including submission of updates, and progress reports | 2% |
| 10 | Participate in database management and data collection process | 10% |
| 11 | Liaise with key stakeholders at participating sites | 3% |

### SELECTION CRITERIA

|  | Essential Requirements |
| --- | --- |
| 1 | Excellent skills in the statistical aspects of trials and data analysis |
| 2 | Excellent project management skills |
| 3 | High level computing skills using statistical software and Microsoft Office programs |
| 4 | Excellent written and verbal communication skills with health professionals and researchers |
| 5 | Ability to work in a multidisciplinary research team |
|  |  |
|  | Desirable Attributes |
| 1 | Previous experience in the conduct of randomised controlled trials |
| 2 | Previous experience in staff management |
| 3 | Previous experience with data management |
| 4 | Nursing and midwifery qualifications |
| 5 | Hospital-based research experience and/or knowledge of the public maternity sector |
