# Supplementary material for: COSMOS: COmparing Standard Maternity care with One-to-one midwifery Support: a randomised controlled trial
Source: BMC Pregnancy Childbirth. 2008 Aug 5;8:35. doi: 10.1186/1471-2393-8-35 (PMC2526977; doi:10.1186/1471-2393-8-35)
Supplement: Additional file 5 — Terms of Reference for Reference group; Safety Committee and Data Monitoring Committee. [file 1471-2393-8-35-S5.doc]

# **Additional file V: Terms of reference for Reference group, Data Monitoring Committee and Safety Committee**

***Caseload midwifery for women at low risk of medical complications: a multi-centre randomised controlled trial (COSMOS)***

***Reference Group***

### *Terms of Reference*

The Caseload midwifery randomised controlled trial (COSMOS) Reference Group is established to bring together a group of people from a range of backgrounds, with relevant expertise and/or an interest in the project to:

- contribute ideas and advice to the research team through all stages of the project
- comment on drafts of materials and resources developed to support the project (eg process and outcome measurement tools/questionnaires, reports)
- provide advice and assistance in promoting the project where appropriate
- participate in discussion of the findings and their implications for health service delivery across Victoria
- assist in developing appropriate strategies for disseminating the findings of the project.

Responsibility for the conduct of the research, analysis of the data and publication of the findings remains with the research team.

Acknowledgement of the involvement of the Reference Group will be made in all publications.

March 21^st^ 2007

**COmparing Standard Maternity care with One to one midwifery Support (COSMOS) Trial-ACTRN012607000073404**

**SAFETY COMMITTEE TERMS OF REFERENCE**

The purpose of this document is to describe the roles and responsibilities of the Safety Committee for the COSMOS trial including the timing of meetings, methods of providing information to and from the Safety Committee, frequency and format of meetings and relationships with other committees.

**Aims:**

To safeguard the interests of trial participants by assessing the safety of participation, and to assist and advise the principal investigators so as to protect the credibility of the trial.

**Relationships:**

The Safety Committee reports to the COSMOS principal investigators. A Data Monitoring Committee (DMC) will be reporting separately on the progress and accruing data from the trial.

**Specific roles of the Safety Committee**

- To review reports of serious adverse outcomes in both trial arms to identify possible contributing factors related to participation in the trial and any association with either trial arm
- To advise the principal investigators if the Safety Committee has concerns about the safety of participants that might require the trial’s early termination
- The adverse events that the Safety Committee will be required to report on are:
  - Perinatal
    - Birth injury requiring admission to special care nursery
    - Birth asphyxia using Apgar < 3 at 5 minutes
    - Neonatal seizures
    - Neonatal encephalopathy
    - Readmission to hospital up to 4 weeks post birth
    - Perinatal death
  - Maternal
    - Maternal death
    - Eclampsia
    - Major postpartum haemorrhage > 1000mls and/or requiring operative procedure and/or blood transfusion
    - Admission to ICU/HDU
    - Prolonged postpartum hospital admission due to serious maternal morbidity
    - Readmission to hospital following birth up to six weeks post birth (excluding mastitis)

**Process**

The research midwives will be extracting data every 2 weeks from the records of women who have given birth. They will report the nominated serious adverse events to the trial coordinator. The trial coordinator will prepare a written report every **500** births for Safety Committee members. The report will include a description of the adverse events and outcomes. All attempts will be made to blind Safety Committee members to group allocation but this may not be possible if an adverse event requires extensive review of the medical record. Written reports will be emailed to all Safety Committee members.

Safety Committee members may request further information regarding an adverse event to assist them with their deliberations.

**Safety Committee Chair**

The Safety Committee members will be asked to elect their own chair.

The chair’s responsibilities will include:

- coordinating email correspondence;
- compiling members’ responses to adverse event reports;
- coordinate (together with the trial coordinator) a review of the medical records of cases that require more extensive review
- providing a written report to the principal investigators;
- maintaining a record of minutes/reports to be forwarded to the trial coordinator on completion of the trial.

**Frequency of reporting**

Safety Committee members will provided with a report at every 500 births summarising the adverse events in both arms of the trial. be notified within 24 hours of adverse events being reported.

**Reporting**

The Safety Committee chair will report the committee’s recommendations/opinions to the COSMOS principal investigators.

**Confidentiality**

The Safety Committee members should destroy their reports after each meeting. The Chair of the Safety Committee will forward copies of all minutes/reports to the trial coordinator on completion of the trial.

**Acknowledgement: The COSMOS trial Safety Committee’s terms of reference have been adapted (in part) from the Charter for Data Monitoring Committees. DAMOCLES Study Group. A proposed charter for clinical trial data monitoring committees: helping them to do their job well. *Lancet 2005; 365:711-22.***

***CO*mparing *S*tandard *M*aternity care with *O*ne to one midwifery *S*upport (COSMOS) Trial-ACTRN012607000073404**

**COmparing Standard Maternity care with One to one midwifery Support (COSMOS) Trial-ACTRN012607000073404**

***DATA MONITORING COMMITTEE TERMS OF REFERENCE***

The purpose of this document is to describe the roles and responsibilities of the independent Data Monitoring Committee (DMC) for the COSMOS trial, including the timing of meetings, methods of providing information to and from the DMC, frequency and format of meetings, statistical issues and relationships with other committees.

**Aims:**

To safeguard the interests of trial participants, assess the efficacy of the intervention during the trial, and monitor the statistical aspects of the trial.

**Terms of reference:**

*The DMC will receive and review the progress and accruing data of the COSMOS trial and provide advice on the conduct of the trial to the principal investigators. The DMC should inform the principal investigators if, in their view:*

1. *the results are likely to convince a broad range of clinicians, including those supporting the trial and the general clinical community, that on balance one trial arm is clearly indicated or contraindicated for all participants or a particular category of participants and there was a reasonable expectation that this new evidence would materially influence patient management;* ***or***
2. *it becomes evident that no clear outcome would be obtained if the trial were to continue to completion*

**Specific roles of the DMC:**

- Monitor recruitment rates and losses to follow-up
- Review interim analyses of data for the primary outcome of interest-caesarean section using criteria agreed to by the principal investigators and the DMC prior to commencement of the trial
- Recommend that the trial continues to recruit participants or that recruitment should be terminated

**DMC relationships:**

The DMC reports to the COSMOS principal investigators. A Safety Committee will be reporting separately on serious adverse events.

**Process:**

During the period of recruitment to the trial, the trial coordinator will coordinate the provision of data including accumulated information relating to recruitment and the primary outcome of interest- caesarean section. The primary outcome data will be provided as raw data with group allocation blinded. The data will be provided in strict confidence to the DMC.

**DMC Chair**

The chair’s responsibilities will include:

- coordinating/chairing teleconference meetings;
- maintaining minutes from meetings;
- providing a written report to the principal investigators;
- providing copies of minutes to the trial coordinator on completion of the trial.

**Meeting frequency:**

It is anticipated that the DMC will meet by teleconference at the beginning of the trial, at one year to monitor recruitment levels and when 1000 births have been reported.

**Reporting**

The DMC will report their recommendations/opinions to the COSMOS principal investigators.

Possible recommendations could include:

- No action needed, trial continues as planned
- Early stopping due, for example, to clear benefit or harm of the treatment

**Confidentiality**

Unless modification or cessation of the protocol is recommended by the DMC, the principal investigators and administrative/research staff (except those who supply the confidential information) will remain unaware of the results of the interim analysis.

The DMC members should destroy their reports after each meeting. Fresh copies of previous reports will be circulated with the newest report before each meeting.

After the trial is reported, the DMC members should destroy all interim reports. The Chair of the DMC will forward copies of all minutes/reports to the trial coordinator on completion of the trial.

**Acknowledgement: The COSMOS Trial DMC terms of reference have been adapted from the Charter for DMCs. DAMOCLES Study Group. A proposed charter for clinical trial data monitoring committees: helping them to do their job well. *Lancet 2005; 365:711-22.***
